# Disruption of ROCK1 gene restores autophagic flux and mitigates doxorubicin-induced cardiotoxicity

**DOI:** 10.18632/oncotarget.24457

**Published:** 2018-02-08

**Authors:** Jianjian Shi, Michelle Surma, Lei Wei

**Affiliations:** ^1^ Riley Heart Research Center, Herman B Wells Center for Pediatric Research, Department of Pediatrics, Indiana University, School of Medicine, Indianapolis, Indiana, USA; ^2^ Department of Cellular and Integrative Physiology, Indiana University, School of Medicine, Indianapolis, Indiana, USA

**Keywords:** Rho kinase, doxorubicin, cardiotoxicity, autophagy, apoptosis

## Abstract

Doxorubicin is among the essential medicines with a wide antitumor spectrum, but its clinical application is limited by its cardiotoxicity. We recently discovered that ROCK1 is a key molecule in mediating cardiac remodeling in response to various stresses. To determine the roles of ROCK1 in doxorubicin cardiotoxicity, we gave three doses of doxorubicin injections to wild type (WT) and ROCK1^−/−^ mice with one week intervals between treatments, the cumulative dose being 24 mg/kg. ROCK1^−/−^ mice exhibited preserved cardiac function, reduced apoptosis, autophagy and fibrosis compared to the WT mice. To further determine the cellular mechanisms, we have examined the role of ROCK1 in cardiomyocytes using cardiomyocyte-specific knockout mice, MHC-Cre/ROCK1^fl/fl^, which partially reproduced the cardioprotective characteristics of ROCK1^−/−^ mice, indicating that ROCK1 in both cardiomyocytes and non-cardiomyocytes mediates doxorubicin cardiotoxicity. To elucidate the molecular mechanisms, a detailed time course study after a single doxorubicin injection at 10 mg/kg was performed in ROCK1^−/−^ and MHC-Cre/ROCK1^fl/fl^ mice. The molecular analysis revealed that both ROCK1^−/−^ and MHC-Cre/ROCK1^fl/fl^ hearts exhibited significant reduction of doxorubicin-induced early responses including increased apoptotic (Bax) and autophagic (p62/SQSTM1 and LC3-II) markers, associated with reduced Beclin 1 phosphorylation on Thr119, supporting reduced Beclin 1-mediated autophagy initiation due to increased association of Beclin 1 with Bcl 2 or Bcl-XL in these hearts compared to the WT or ROCK1^fl/fl^ mice. These results support that ROCK1 deficiency is cardioprotective against doxorubicin-induced cardiotoxicity at least in part through reducing Beclin 1-mediated autophagy initiation in cardiomyocytes and restoring autophagic flux to ameliorate doxorubicin cardiotoxicity.

## INTRODUCTION

The anthracyclines, mainly doxorubicin, are among the most widely used and successful drugs to treat a wide spectrum of hematologic malignancies and solid tumors. But anthracycline-induced dose-dependent and cumulative cardiotoxic side effects cause persistent and progressive damage to the cardiovascular system [1–4]. Therefore, their usefulness in cancer treatment is significantly compromised. Children and adolescents are particularly vulnerable to anthracycline cardiotoxicity. About half of the young adult survivors of childhood cancer have received anthracyclines in their treatment [4–7]. Regardless of the severe cardiotoxicity, anthracyclines are still irreplaceable in cancer therapeutic schemes.

Although intensive investigations have improved our insights on anthracycline cardiotoxicity, the underlying mechanisms have not yet been completely elucidated; therefore new strategies providing effective prevention of cardiac side effects continue being in great demand. Various mechanisms have been proposed; they include, but are not limited to: free radical-induced oxidative stress, damage to nuclear DNA, dysregulation of calcium handling and cellular contractility, suppression of transcription factors that regulate cell survival and sarcomere protein synthesis, disruption of sarcomere stability, and mitochondrial dysfunction in cardiomyocytes [8–13]. Most of these cellular events eventually contribute to cardiomyocyte death. Indeed, there is accumulating experimental evidence that cardiomyocyte apoptosis [13–15] and impaired autophagic function [16–21] play important roles in doxorubicin-induced cardiomyopathy.

Autophagy is a cell survival mechanism aimed at maintaining cell and tissue homeostasis under normal as well as stress conditions, including nutrient starvation, metabolic intervention, energy machinery dysfunction, and oxidative stress. Autophagy, characterized by the formation of autophagosomes, is an orchestrated process involving several steps: initiation, nucleation, elongation, maturation and degradation. Numerous studies have demonstrated that doxorubicin-induced cardiac injury is associated with dysregulation in autophagic function [16–21]. Recent studies support the notion that doxorubicin-induced cardiotoxicity causes an over-activation of autophagy initiation due to increased cellular damage while preventing autophagy completion due to deleterious effects on lysosomes, which results in the accumulation of un-degraded protein aggregates or damaged organelles [16–21]. Attenuating autophagic dysregulation in doxorubicin-treated hearts represents an attractive strategy to prevent or mitigate doxorubicin-induced cardiomyopathy [16–21].

ROCKs are central regulators of the actin cytoskeleton downstream of the small GTPase RhoA [22–31]. The two ROCK isoforms, ROCK1 and ROCK2, are highly homologous with an overall amino acid sequence identity of 65% [22–24]. We recently found that ROCK1 is a key molecule in mediating apoptotic signaling in cardiomyocytes under pressure overload and in genetically-induced pathological cardiac hypertrophy [32–36]. Using mouse embryonic fibroblasts as an *in vitro* system, we observed that ROCK1 deficiency has a unique protective benefit of preserving actin cytoskeleton stability, which acts additively with antioxidant treatment to suppress excessive production of doxorubicin-induced reactive oxygen species and apoptosis [37–40]. The present study is focused on the *in vivo* role of ROCK1 in mediating doxorubicin cardiomyopathy, particularly on doxorubicin-induced autophagy dysregulation. We used both systemic ROCK1 deficient mice (ROCK1^−/−^) and cardiomyocyte-specific ROCK1 knockout mice using MHC-Cre mice [41] crossed into ROCK1^fl/fl^ in this study. In addition to demonstrating an *in vivo* role of ROCK1 in mediating doxorubicin cardiotoxicity and cardiomyocyte apoptosis, we have uncovered a role for ROCK1 in mediating doxorubicin-induced dysregulation of autophagic flux in cardiomyocytes, possibly through promoting Beclin 1-mediated autophagy initiation, further supporting that ROCK1 represents a potential therapeutic target to prevent chemotherapeutic drug doxorubicin-induced heart failure.

## RESULTS

### ROCK1 deficient mice are protected from doxorubicin cardiotoxicity associated with attenuation of apoptosis and autophagy dysregulation

**Figure 1:**
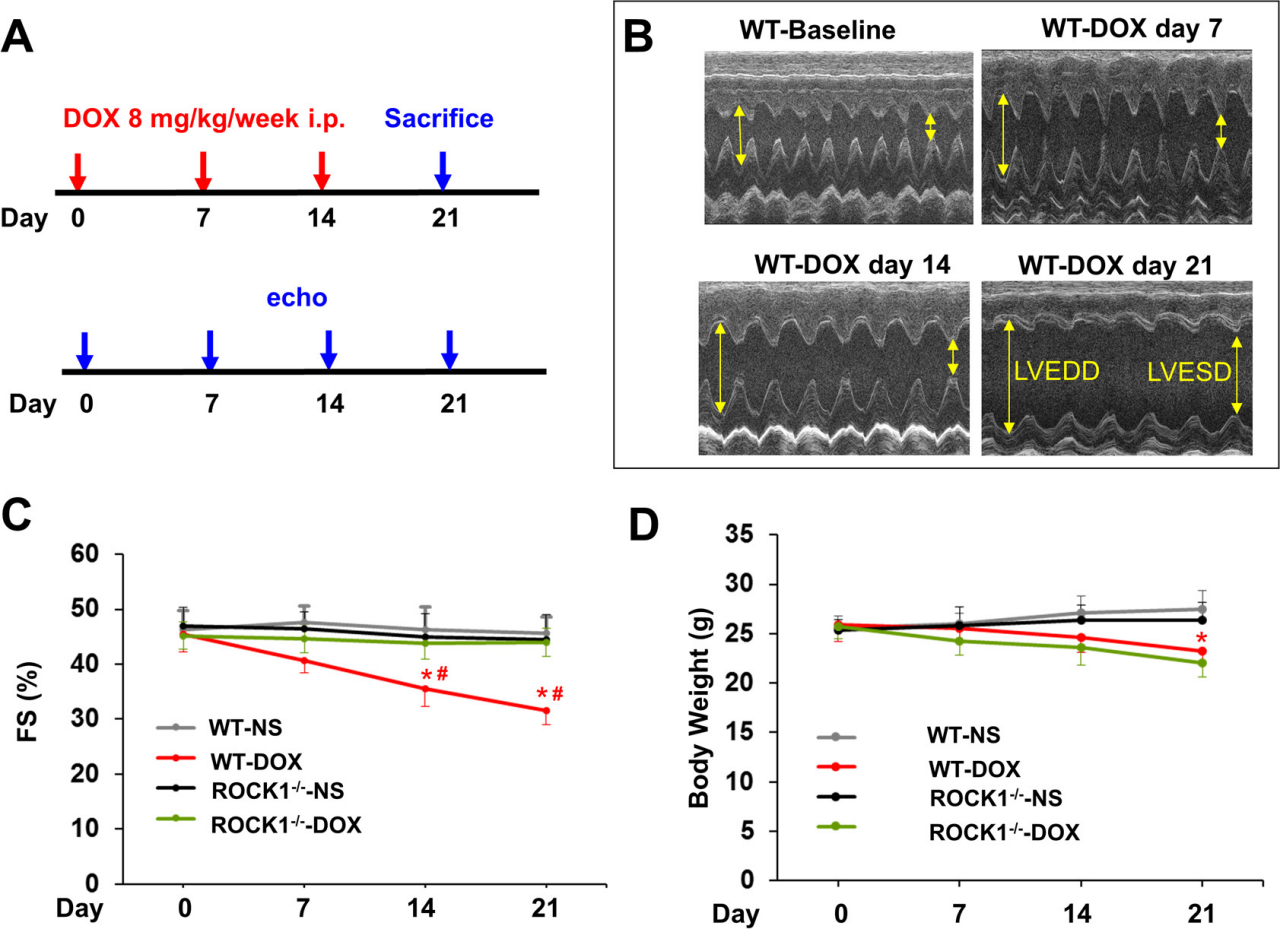
Time course study of doxorubicin cardiotoxicity **(A)**. Schematic of serial doxorubicin administration protocol. FVB WT and ROCK1 deficient mice 8 to 9 weeks old received three serial injections weekly of normal saline (NS) or doxorubicin (DOX) (8 mg/kg). Mice were sacrificed on day 21 after the initial injection. **(B)**. Representative short-axis echocardiograms from WT mice before each injection, and in 1 week after the 3^rd^ dose. **(C)**. Cardiac function in WT mice, but not in ROCK1 deficient mice, was significantly impaired on day 14, 1 week after the 2^nd^ dose. FS, fractional shortening. **(D)**. DOX significantly affected body weight on day 21 after the initial injection in both WT and ROCK1 deficient mice. N = 10-15 in each group. ^*^p < 0.05 for DOX-injected vs. NS-injected mice. ^#^*p* < 0.05 for DOX-treated ROCK1 deficient mice vs. DOX-injected WT mice.

**Figure 2:**
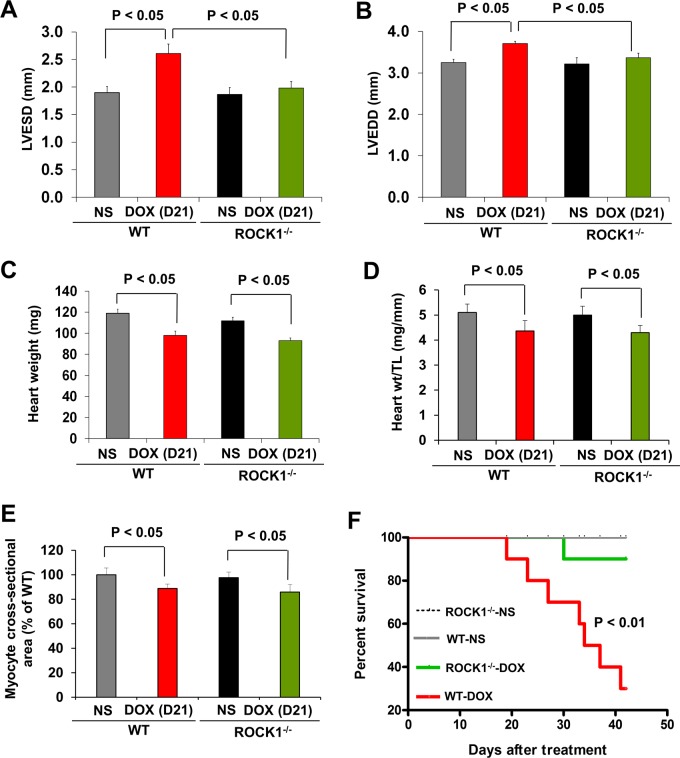
ROCK1 deficient mice are protected from doxorubicin cardiotoxicity **(A-B)**. Echocardiography analysis of WT and ROCK1 deficient mice on day 21 after the initial injection. Cardiac dimension were preserved in DOX-treated ROCK1 deficient mice compared with WT mice. LVESD, left ventricular end systolic dimension; LVEDD, left ventricular end diastolic dimension. N = 8-10 in each group. **(C-D)**. Quantitative analysis of heart weight (C), heart weight (Heart wt)/tibial length (TL) ratios from WT and ROCK1 deficient mice on day 21 after the initial injection. **(E)**. Quantitative analysis of cardiac myocyte area measured from laminin stained sections. Each column represents results obtained from approximately 200 myocytes from at least four hearts per group. ROCK1 deficiency doesn’t prevent DOX-induced reduction in heart weight and cardiomyocyte size. **(F)**. Kaplan-Meier survival curves (over 6 weeks after the initial DOX injection) indicate that the mortality rate was significantly lower in the ROCK1 deficient mice than that in WT mice. N = 10 in each group. *p* < 0.01 for DOX-injected ROCK1 deficient vs. DOX-injected WT mice.

Mice 8 to 9 weeks old were injected intraperitoneally with doxorubicin at 8 mg/kg or normal saline (NS) once weekly for 3 consecutive weeks, the cumulative dose totaling 24 mg/kg (Figure 1A). This treatment is based on the approach previously described [15, 42], which causes progressive cardiac dysfunction within three weeks after the initial injection, a scenario that mimics early-onset doxorubicin-induced cardiotoxicity in humans. Echocardiograms revealed that doxorubicin treatment resulted in reproducible and progressive left ventricular (LV) dilation in WT mice as evidenced by increased end systolic dimension (LVESD) and end diastolic dimension (LVEDD; Figure 1B) over 3 weeks after initiation of the treatment. Consistent with cardiac dilation, LV contractile function (as measured by LV fractional shortening, FS) was reduced in doxorubicin-treated WT mice (Figure 1C, 2A, 2B). Doxorubicin had a significant impact on body weight (Figure 1D), heart weight (Figure 2C, 2D), and cardiomyocyte size (Figure 2E). For subsequent studies of phenotype, we focused on day 21 after the initial injection, when no significant animal death was detected in WT mice (Figure 2F).

To elucidate the role of ROCK1 in doxorubicin cardiotoxicity, ROCK1^−/−^ mice were treated with doxorubicin in parallel to the WT mice. In contrast to the WT mice, doxorubicin-induced LV dilation was blocked in ROCK1^−/−^ mice, and cardiac function was preserved (Figure 1C, 2A, 2B) over 3 weeks after initiation of the treatment. Doxorubicin had a similar impact on body weight (Figure 1D), heart weight (Figure 2C, 2D), and cardiomyocyte size (Figure 2E) in ROCK1^−/−^ mice compared to WT mice, indicating that the protective effects of ROCK1 deletion are not mediated by inhibition of weight loss and cardiomyocyte atrophy. Kaplan-Meier survival curves over 6 weeks after the initial doxorubicin injection indicate that the mortality rate was significantly lower in the treated ROCK1^−/−^ group (about 10%) than that in the treated WT group (about 70%) (Figure 2F). In addition, all treated WT mice died within 3 months after starting doxorubicin treatment, whereas the mortality rate of treated ROCK1 deficient mice reached to 55% at 3 months and to 70% at 6 months. These results support that ROCK1 deficiency reduces not only cardiac toxicity but also systemic toxicity caused by doxorubicin.

**Figure 3:**
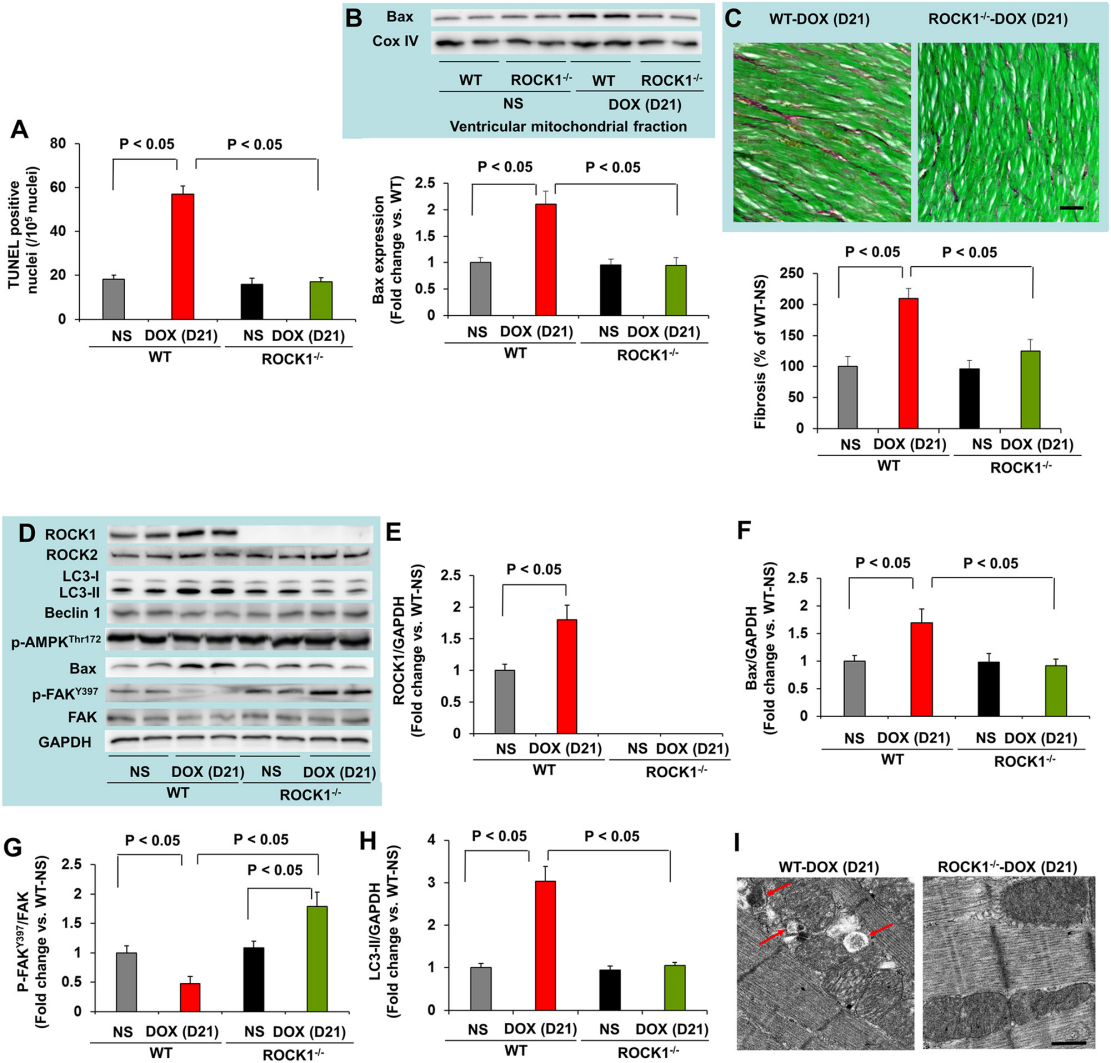
ROCK1 deletion inhibited doxorubicin-induced apoptosis, fibrosis and autophagy **(A)**. Quantification of total TUNEL positive nuclei per 10^5^ total nuclei in ventricular myocardium from WT and ROCK1 deficient hearts on day 21 after the initial injection. N = 4-6 in each group. **(B)**. Representative images (top) of Western blot analysis of Bax and Cox IV in mitochondrial fraction of ventricular homogenates from WT and ROCK1 deficient hearts. Quantitative analysis (bottom) of immunoreactive bands of Bax (N = 4-6 in each group) expressed as fold change relative to NS-treated WT group. **(C)**. Representative heart sections (top) stained with picrosirius red/Fast green (scale bar, 50 μm) showing collagen deposition. Quantitative analysis (bottom) of the collagen deposition in WT and ROCK1 deficient hearts, expressed as percentage change relative to NS-treated WT hearts. Four to six hearts in each group. At least 10 randomly chosen high power fields per section and four transverse sections from each heart, sampled from the midpoint between the apex and base, were analyzed. **(D)**. Representative images of Western blot analysis of ROCK1, ROCK2, LC3, Beclin 1, p-AMPK-Thr172, Bax, FAK and p-FAK-Tyr397 in ventricular homogenates of WT and ROCK1 deficient hearts on day 21 after the initial injection. **(E-H)** Quantitative analysis of immunoreactive bands of ROCK1 (E), Bax (F), p-FAK-Tyr397/FAK (G) and LC3-II (H). N = 4-6 in each group. **(I)**. Representative transmission electron microscopy images of WT and ROCK1 deficient hearts on day 21 after the initial injection. DOX-treated WT heart showed increased numbers of autophagic vacuoles (red arrows). Scale bar, 0.5 μm.

Since doxorubicin treatment induced significant increases in cardiomyocyte and non-cardiomyocyte apoptosis [14], we assessed apoptosis by TUNEL staining. The number of TUNEL positive cardiac cells was significantly increased in doxorubicin-treated WT mouse hearts (Figure 3A), and was associated with increased mitochondrial translocation of Bax (Figure 3B). Cardiac fibrosis was also increased in doxorubicin-treated WT mice (Figure 3C). However, these characteristics of doxorubicin cardiotoxicity were effectively blocked in ROCK1^−/−^ mice (Figure 3A–3C). Molecular analysis also revealed increased ROCK1 expression in doxorubicin-treated WT mice, associated with increased Bax levels and decreased focal adhesion kinase (FAK) phosphorylation (Figure 3D–3G), further supporting a role of ROCK1 in doxorubicin-induced cardiotoxicity. During autophagy, the cytosolic form of microtubule-associated protein 1 light chain 3 (LC3-I) is conjugated to phosphatidylethanolamine to form LC3-II, which is recruited to autophagosomal membranes [43]. Interestingly, the levels of LC3-II were increased by about 3-fold in doxorubicin-treated WT hearts but not in ROCK1^−/−^ hearts (Figure 3D, 3H), suggesting that ROCK1 deletion prevents the dysregulation of autophagy induced by doxorubicin. Consistent with this notion, transmission electron microscopy revealed more accumulation of autophagosomes in doxorubicin-treated WT mouse hearts than in ROCK1^−/−^ hearts (Figure 3I). Moreover, this increased LC3-II accumulation was not accompanied by increased levels of Beclin 1 and AMP-activated protein kinase (AMPK) phosphorylation (Figure 3D), suggesting that the accumulation of autophagosomes was not due to the activation of either AMPK-mediated autophagy or a general up-regulation of the autophagy system.

### Cardiomyocyte-specific ROCK1 knockout mice are also protected from doxorubicin cardiotoxicity

**Figure 4:**
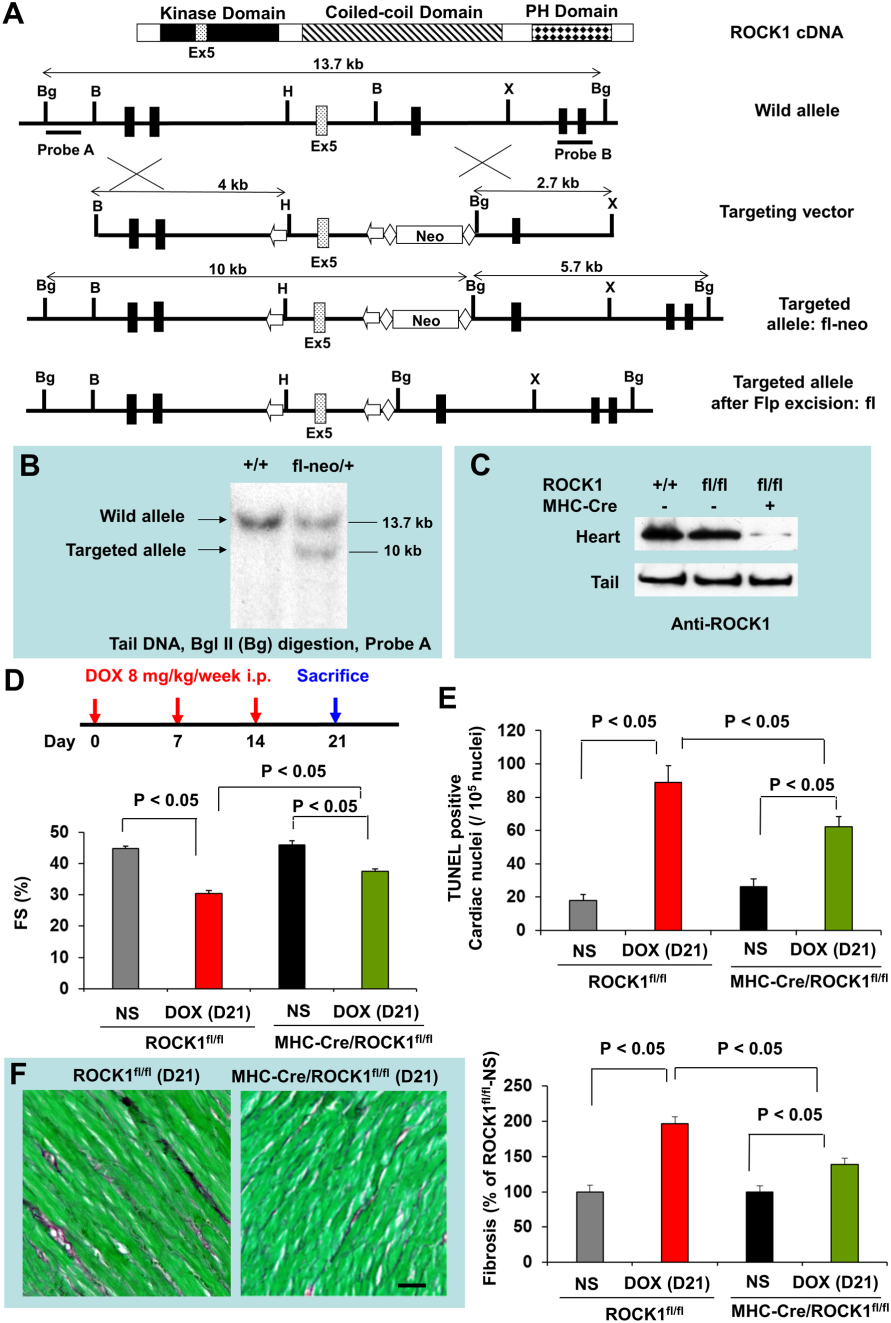
Cardiomyocyte-specific ROCK1 deletion also inhibited doxorubicin-induced cardiac dysfunction, apoptosis and fibrosis **(A)**. Schematic illustration of the gene targeting strategy used for conditional disruption of the ROCK1 gene. Schematic representation of the domain structure of ROCK1 indicates the position of exon 5. A fragment of the ROCK1 allele that includes exons 3-6 is shown. A targeting vector was constructed containing loxP sites (arrows) flanking exon 5 of ROCK1 and Frt sites (diamonds) flanking the PGK-Neo cassette. The diagrams also indicate the positions of genomic probes used for distinguishing WT and the targeted alleles by Southern blot analysis and the positions of the restriction enzymes sites for *Bam* H I (B), *Bgl* II (Bg), *Xba* I (X), and *Hind* III (H). We first obtained germline transmission of the ROCK1^fl-neo^ allele, which contains the loxP-flanked exon 5 and Neo cassette. The Neo cassette was removed via the Flp-Frt system by crossing ROCK1^fl-neo/fl-neo^ mice with Flp mice, generating ROCK1^fl/+^ mice. **(B)**. Southern blot analysis of genomic DNA obtained from the tail of WT and ROCK1^fl-neo/+^ mice. **(C)**. Western blot analysis of ROCK1 levels in the heart and tail of WT, ROCK1^fl/fl^ and MHC-Cre/ROCK1^fl/fl^ mice, showing about 80% reduction of ROCK1 expression in the heart samples of the MHC-Cre/ROCK1^fl/fl^ mice compared with WT or ROCK1^fl/fl^ mice, but not in the tail samples of these mice. Residual ROCK1 expression in the heart is due to the presence of ROCK1 in other cell types in hearts (e.g., fibroblasts, vascular endothelial cells, and inflammatory cells). **(D)**. Cardiomyocyte-specific ROCK1 knockout mice (MHC-Cre/ROCK1^fl/fl^) and ROCK1^fl/fl^ mice 8 to 9 weeks old received three serial injections weekly of NS or DOX (8 mg/kg). Cardiac function was measured by echocardiography analysis on day 21 after the initial injection. **(E-F)**. Quantitation of total TUNEL positive nuclei per 10^5^ total nuclei (E) and the collagen deposition (F, scale bar, 50 μm) in ventricular myocardium from MHC-Cre/ROCK1^fl/fl^ and ROCK1^fl/fl^ mice hearts on day 21. N = 4-6 in each group.

To determine if ROCK1 in cardiomyocytes has an important role in doxorubicin cardiotoxicity, we generated cardiomyocyte-specific ROCK1 knockout mice. In myosin heavy chain (MHC)-Cre/ROCK1^fl/fl^ mice, the ROCK1 protein is truncated from residue 137 to the end of the protein (Figure 4A–4C) only in cardiomyocytes. Similar to ROCK1^−/−^ mice in which the ROCK1 protein is also truncated from residue 180 to the end of the protein in all cells [32], the conditional targeting approach results in the removal of a large portion of the kinase domain and amino acids from the coiled-coil and the PH domains (Figure 4A). We have noted that MHC-Cre alone had no significant cardiotoxic effects at baseline as no significant morphological or molecular differences in apoptosis and autophagy markers were observed in MHC-Cre mice compared to WT mice at 8 to 9 weeks old (data not shown). We first examined the cardiac response to doxorubicin in MHC-Cre/ROCK1^fl/fl^ mice 8 to 9 weeks old subjected to the three-injection treatment compared to ROCK1^fl/fl^ mice (Figure 4D–4F). Similar to the finding from WT mice, doxorubicin induced cardiac dysfunction (Figure 4D) and increased TUNEL positivity (Figure 4E) and cardiac fibrosis (Figure 4F) in ROCK1^fl/fl^ hearts. However, these cardiotoxic events were significantly reduced in MHC-Cre/ROCK1^fl/fl^ mice (Figure 4D–4F), supporting that ROCK1 in cardiomyocytes contributes to doxorubicin-induced cardiotoxicity. We have also noted the differences between systemic ROCK1 knockout and MHC-Cre/ROCK1^fl/fl^ mice; in the former there were no significant changes in cardiac function, TUNEL positivity and fibrosis after doxorubicin treatment (Figure 1-3), but these parameters were significantly changed in the latter (Figure 4). Though these increases are at lower levels in comparison with ROCK1^fl/fl^ mice, it indicates that ROCK1 in other non-cardiomyocyte cardiac cells also contributes to the cardiac response to doxorubicin.

### Systemic ROCK1 deletion ameliorates doxorubicin-induced autophagy dysregulation through decreasing Beclin 1-mediated autophagosome formation

**Figure 5:**
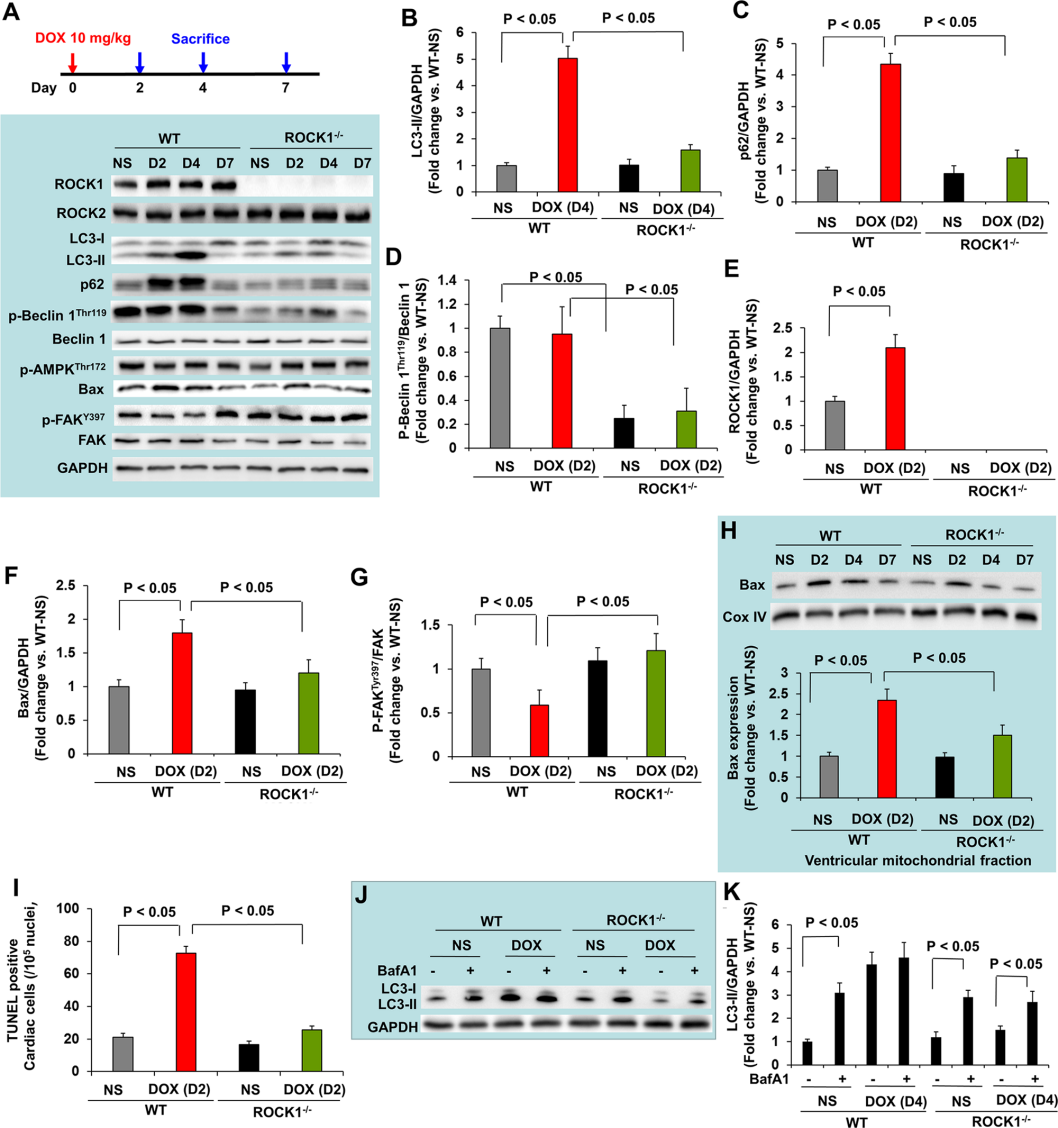
ROCK1 deletion inhibited the early onset of doxorubicin-induced autophagy dysregulation and apoptosis **(A)**. Schematic of single DOX administration protocol (top). Mice 8 to 9 weeks old received one injection of NS or DOX (10 mg/kg). Mice were sacrificed on day 2, 4 and 7 after the injection. Representative images (bottom) of Western blot analysis of ROCK1, ROCK2, LC3, p62, Beclin 1, p-Beclin 1-Thr119, p-AMPK-Thr172, Bax, FAK and p-FAK-Tyr397 in ventricular homogenates of WT and ROCK1 deficient hearts on day 2, 4 and 7 after a single DOX injection. **(B-G)**. Quantitative analysis of immunoreactive bands of LC3-II on day 4 (B), p62 on day 2 (C), p-Beclin1-Thr119/Beclin 1 on day 2 (D), ROCK1 on day 2 (E), Bax on day 2 (F), p-FAK-Tyr397/FAK on day 2 (G) after single DOX injection. N = 4-6 in each group. **(H)**. Representative images (top) of Western blot analysis of Bax and Cox IV in the mitochondrial fraction of ventricular homogenates from WT and ROCK1 deficient hearts. Quantitative analysis (bottom) of immunoreactive bands of Bax on day 2 after single DOX injection (N = 4-6 in each group) expressed as fold change relative to NS-treated WT group. **(I)**. Quantification of total TUNEL positive nuclei per 10^5^ total nuclei in ventricular myocardium from WT and ROCK1 deficient hearts on day 2 after single DOX injection. N = 4-6 in each group. **(J-K)**. Mice 8 to 9 weeks old received one injection of saline or DOX (10 mg/kg). On day 4 after the injection, mice received one injection of bafilomycin A1 at 1.5 mg/kg 2 hours before they were sacrificed. Representative images (J) and quantitative analysis (K) of Western blot analysis of LC3-II in ventricular homogenates of WT and ROCK1 deficient hearts. N = 4-6 in each group expressed as fold change relative to NS-treated WT group.

To determine the molecular mechanisms underlying the protective effects of ROCK1 deletion, we evaluated the time course of doxorubicin-induced apoptosis and autophagy dysregulation and performed molecular analysis at day 2, 4 and 7 after a 10 mg/kg single dose injection. The maximal increase in the autophagy marker, LC3-II, occurred on day 4 (Figure 5A, 5B). This increased level of LC3-II could derive from an over-activation of autophagy initiation or from the prevention of autophagy completion due to deleterious effects on lysosomes. To distinguish these two possibilities, we measured the accumulation of p62/sequestosome 1 (SQSTM1), another protein marker of autophagy, which was increased at day 2, preceding maximal LC3-II accumulation (Figure 5A, 5C). Since p62/SQSTM1 is an adaptor protein that is cleared by autophagy [43], the increased accumulation suggests that the degradation process of autophagosomes was impaired in doxorubicin treated hearts. The increased LC3-II and p62/SQSTM1 accumulations were not associated with increases in Beclin 1 levels or in phosphorylation of AMPK (Figure 5A), suggesting that the accumulation of autophagosomes was not due to the activation of AMPK-mediated autophagy or a general up-regulation of the autophagy system. Interestingly, ROCK1 deletion abolished doxorubicin-induced increases in LC3-II and p62/SQSTM1 levels (Figure 5A–5C) at these early time points, indicating that ROCK1 deletion improves autophagic flux.

Regarding the molecular changes implicated in apoptosis, increases in ROCK1 and Bax expression and decreases in phosphorylation of FAK were observed at day 2 (Figure 5A, 5E-5G), coinciding with increased mitochondrial translocation of Bax (Figure 5H) and increased TUNEL positivity (Figure 5I). A trend toward increased Bax, mitochondrial Bax and TUNEL positivity was noticed in doxorubicin-treated ROCK1^−/−^ hearts compared to NS-treated hearts, but the differences were not statistically significant (Figure 5A, 5F, 5H, 5I). These results indicate that both apoptosis and the impairment of autophagic flux occur concurrently at early time points after doxorubicin treatment.

To further investigate the mechanisms underlying the beneficial effects of ROCK1 deletion in improving autophagic flux, we examined the phosphorylation levels of Beclin 1 at Thr119 (Figure 5A, 5D). It has been reported that ROCK1-mediated Beclin 1 phosphorylation promotes the dissociation of Beclin 1 from Bcl 2 resulting in increased autophagosome formation induced by nutritional stress [44]. In ROCK1^−/−^ hearts, the levels of p-Beclin 1^Thr119^ were reduced at all time points tested including the baseline condition, suggesting that ROCK1 deletion inhibits Beclin 1-mediated autophagosome formation. To further demonstrate that ROCK1 deletion can improve autophagic flux, WT and ROCK1^−/−^ mice were treated with either NS or doxorubicin, and mice were euthanized on day 4, 2 hours after an injection of bafilomycin A1 which prevents the fusion of autophagosome with the lysosome and blocks autophagic flux. Bafilomycin A1 injection in control animals resulted in a significant increase in LC3-II levels, reflecting cardiac autophagic flux under basal conditions (Figure 5J, 5K). Bafilomycin A1 injection in doxorubicin-treated WT mice showed no increase in LC3-II levels, indicating a blockage in autophagic flux. Since the absence of added effects of bafilomycin A1 could be the result of saturated activity of LC3-II on day 4 when LC3-II reached maximal levels (Figure 5A), bafilomycin A1 injection was also performed on day 2 in the WT hearts and no added effects were observed (data not shown), further supporting a blockage in autophagic flux in WT hearts. In contrast to WT mice, bafilomycin A1 injection in doxorubicin-treated ROCK1^−/−^ mice on day 4 resulted in a significant increase in LC3-II levels (Figure 5J, 5K), indicating that autophagic flux was maintained in ROCK1^−/−^ mice after doxorubicin treatment. Consistent with a previous report showing that mice with a heterozygous deletion of Beclin 1 are cardioprotective to doxorubicin through reducing the initiation of autophagy, which allows for the preservation of autophagic flux in the presence of impaired lysosome function by doxorubicin [16], our results demonstrate that attenuated autophagy initiation in ROCK1^−/−^ mice results in limiting the accumulation of unprocessed autolysosomes and ameliorating doxorubicin cardiotoxicity.

### Cardiomyocyte ROCK1 deletion also ameliorates doxorubicin-induced autophagy dysregulation through decreasing Beclin 1-mediated autophagosome formation

**Figure 6:**
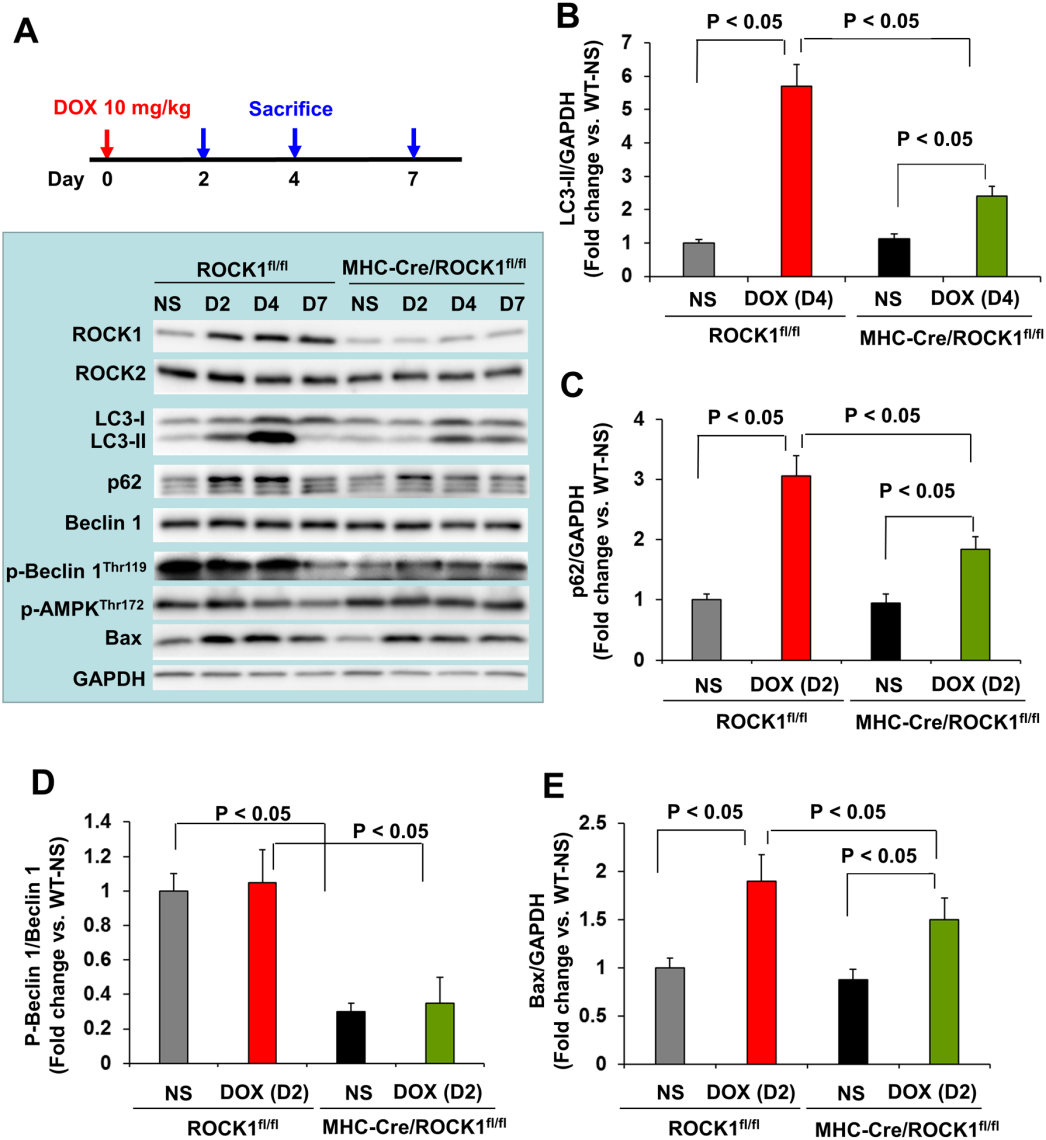
Cardiomyocyte-specific ROCK1 deletion also inhibited the early onset of doxorubicin-induced autophagy dysregulation **(A)**. Cardiomyocyte-specific ROCK1 knockout mice (MHC-Cre/ROCK1^fl/fl^) and ROCK1^fl/fl^ mice 8 to 9 weeks old received one injection of NS or DOX (10 mg/kg). Mice were sacrificed on day 2, 4 and 7 after the injection and Western blot analysis was performed with ventricular homogenates. Representative images of Western blot analysis of ROCK1, ROCK2, LC3, p62, Beclin 1, p-Beclin 1-Thr119, p-AMPK-Thr172 and Bax in ventricular homogenates on day 2, 4 and 7 after single DOX injection. **(B-E)**. Quantitative analysis of immunoreactive bands of LC3-II on day 4 (B), p62 on day 2 (C), p-Beclin1-Thr119/Beclin 1 on day 2 (D) and Bax on day 2 (E) after single DOX injection. N = 4-6 in each group.

Finally, to determine the molecular mechanisms in cardiomyocytes, molecular analysis was performed at day 2, 4 and 7 after a single dose injection in ROCK1^fl/fl^ and MHC-Cre/ROCK1^fl/fl^ mice (Figure 6). Similar to the WT mice, increases in ROCK1 expression were observed from day 2 in ROCK1^fl/fl^ mice (Figure 6A). However, deletion of ROCK1 in cardiomyocytes abolished this molecular change (Figure 6A) indicating that the doxorubicin-induced ROCK1 up-regulation occurs in cardiomyocytes. The time course showing doxorubicin-induced increases in autophagy markers (LC3-II, p62/SQSTM1) and an apoptosis marker (Bax) was also similar in ROCK1^fl/fl^ mice compared to the WT mice (Figure 6A–6CA, 6E), and these marker changes were not associated with increases in Beclin 1 levels or in phosphorylation of AMPK (Figure 6A). ROCK1 deletion in cardiomyocytes significantly reduced the induction of these autophagy and apoptosis markers at the indicated early time points (Figure 6A–6CA, 6E). However, differences existed between systemic ROCK1 knockout and MHC-Cre/ROCK1^fl/fl^ mice: even though they were at lower levels compared to ROCK1^fl/fl^ mice, significant increases of autophagy and apoptosis markers were noted in doxorubicin treated MHC-Cre/ROCK1^fl/fl^ hearts (Figure 6), but not in ROCK1^−/−^ hearts (Figure 5), indicating that ROCK1 in other non-cardiomyocyte cardiac cells also contributes to doxorubicin-induced apoptosis and autophagy dysregulation. Notably, ROCK1-mediated Beclin 1 phosphorylation occurs in cardiomyocytes revealed by reduced levels of p-Beclin 1^Thr119^ at all tested time points in MHC-Cre/ROCK1^fl/fl^ hearts compared to the ROCK1^fl/fl^ hearts (Figure 6A, 6D). Our results indicate that ROCK1 deletion in cardiomyocytes inhibits Beclin 1-mediated autophagosome formation, resulting in reduced autophagy initiation and subsequently reduced accumulation of unprocessed autolysosomes, consequently contributing to the amelioration of doxorubicin-induced cardiotoxicity.

## DISCUSSION

The present study has examined the beneficial effects of systemic ROCK1 deficiency and cardiomyocyte-specific ROCK1 deficiency in the context of doxorubicin-induced cardiotoxicity: both systemic and cardiomyocyte-specific ROCK1 deficiency can provide cardioprotection. More specifically, the beneficial effects of ROCK1 deficiency in reducing cell death due to apoptosis and autophagy are consistent with improved cardiac structure and function. Although the favorable effects of ROCK1 deficiency on cardiac remodeling in other pathological cardiac hypertrophy models have been reported, including pressure overload and genetically-induced pathological cardiac hypertrophy [32–36], the current study has further extended the cardioprotective roles of ROCK1 deficiency against doxorubicin cardiotoxicity, and discovered a role for ROCK1 in mediating doxorubicin-induced dysregulation of autophagic flux in cardiomyocytes.

Doxorubicin-induced increases in autophagosome formation and elevated autophagic markers (p62/SQSTM1 and LC3-II) can be detected within two days after a single dose of doxorubicin, and are also detectable over three weeks with three doses of doxorubicin. ROCK1 deficient mouse hearts exhibited reduced accumulation of autophagosomes and reduced autophagic markers at early time points and during the doxorubicin-induced cardiac remodeling period (Figures 3, 5). Beclin 1 phosphorylation on Thr119 has previously been shown to be involved in the dissociation of Beclin 1 from Bcl 2 [44] or Bcl-X_L_ [45] resulting in Beclin 1-mediated autophagy initiation. We observed in the current study that Beclin 1 phosphorylation was reduced in both systemic and cardiomyocyte-specific ROCK1 knockout mouse hearts at baseline and after doxorubicin treatment (Figures 5, 6), supporting the notion that Beclin 1-mediated autophagy initiation is suppressed by ROCK1 deficiency. This observation is consistent with previously reported protective effects against doxorubicin cardiotoxicity in heterozygous Beclin 1 knockout mice [16].

Numerous studies have demonstrated that doxorubicin-induced cardiac injury is associated with a dysregulation of autophagic function [16–21]. However, findings have been largely inconsistent regarding how doxorubicin dysregulates cardiac autophagy and whether an increase or decrease in autophagy is responsible for doxorubicin cardiotoxicity [16–21]. Recent studies support the notion that doxorubicin leads to an over-activation of autophagy initiation while at the same time preventing autophagy completion due to deleterious effects on lysosomes, resulting in the accumulation of un-degraded protein aggregates or damaged organelles which then contribute to doxorubicin cardiotoxicity [20, 21]. Our present observations indicate that the increase of p62/SQSTM1 levels preceded the maximal increases in LC3-II levels, and doxorubicin-treated WT mice showed no increase in LC3-II levels associated with bafilomycin A1 treatment (Figure 5), pointing towards the disruptions of autophagic flux by doxorubicin. Similar to a heterozygous deletion of Beclin 1, which decreases autophagy initiation resulting in reduced accumulation of unprocessed autolysosomes to ameliorate doxorubicin cardiotoxicity [16], ROCK1 deficiency has a protective role against doxorubicin-induced cardiotoxicity associated with reducing Beclin 1-mediated autophagy initiation and maintaining autophagic flux in cardiomyocytes, thereby reducing the increased demand from an inefficient clearance of autophagosomes by damaged lysosomes.

The contribution of doxorubicin-induced apoptosis to cardiotoxicity has been extensively studied [13–15]. Consistent with this view, we observed that doxorubicin induced a 3 to 4-fold increase in TUNEL positivity in both WT and ROCK1^fl/fl^ hearts associated with the increases in Bax expression and Bax translocation to the mitochondria (Figure 3-6). The increase of the apoptotic marker, Bax, occurred concomitantly with the increase of autophagic markers LC3-II and p62/SQSTM1, indicating that both apoptotic upregulation and autophagic dysregulation are triggered by doxorubicin during the early stages of toxicity. Compared to the systemic ROCK1 deficiency, the cardiomyocyte-specific ROCK1 knockout only partially reproduced the beneficial phenotypes, including improvement in cardiac function, attenuation of apoptotic upregulation and autophagic dysregulation, and reduction in cardiac fibrosis; these observations have demonstrated that ROCK1 in both cardiomyocytes and in non-cardiomyocytes contributes to doxorubicin-induced cardiotoxicity. This notion is consistent with our previous finding that the majority of the apoptosis triggered by doxorubicin treatment occurred in non-cardiomyocytes [14]. In addition to suppressing Beclin 1 phosphorylation to inhibit Beclin 1-mediated autophagy initiation in cardiomyocytes, other mechanisms may also contribute to the cardiac effects of ROCK1 deletion, including improved actin cytoskeleton stability and cell adhesion resulting in enhanced survival signaling. In support of improved cell adhesion, FAK phosphorylation was preserved or enhanced in ROCK1 deficient hearts after doxorubicin treatment (Figure 3, 5). This *in vivo* observation is consistent with previous studies using cultured mouse embryonic fibroblasts in which ROCK1 deficiency, through reducing actin cytoskeleton remodeling, acts additively with antioxidant treatment to suppress excessive production of doxorubicin-induced reactive oxygen species and apoptosis [37–40].

Reported by an *in vivo* study in rats using fasudil, a ROCK inhibitor for both ROCK1 and ROCK2 which also inhibits some other kinases, it was shown that ROCK has a role in mediating doxorubicin-induced cardiotoxicity in rats [46]. Similar to that study, we observed that the doxorubicin treatment increased ROCK1 expression in mouse hearts not only at early time points but also during the doxorubicin-induced cardiac remodeling period (Figures 3, 5, 6). Cardiomyocyte-specific ROCK1 deletion completely suppressed this molecular event, indicating that doxorubicin-induced ROCK1 upregulation occurs largely in cardiomyocytes (Figure 6A). Although fasudil can inhibit doxorubicin-induced apoptosis in rat hearts [46], the effect of the inhibitor on doxorubicin-induced autophagy was not explored. The current study, using a genetic approach, provides novel cellular and mechanistic insights such as promoting Beclin 1-mediated autophagy initiation underlying the detrimental roles of ROCK1 in doxorubicin-induced cardiotoxicity.

Numerous studies have demonstrated that RhoA/ROCK signaling contributes to autophagy, however both positive [47, 48] and negative [44, 49] effects of ROCK inhibition on autophagy have been reported. Treatment with the ROCK inhibitor Y27632 increased the degradation of mutant Huntington protein via proteasome degradation and autophagy in mouse neuroblastoma cell lines [48]. In addition, ROCK inhibition has been linked to an increased autophagy response to starvation or rapamycin treatment and associated with the formation of enlarged early autophagosomes and late degradative autolysosomes in human embryonic kidney 293 cells [47]. In contrast, ROCK inhibition impaired the starvation-mediated autophagic response in HeLa cells and CHO cells through its inhibitory effect on actin cytoskeleton formation which participates in the initial membrane remodeling at very early stages of autophagosome formation [49]. Moreover, systemic ROCK1 deletion impaired the starvation-mediated autophagic response in mouse hearts through inhibiting Beclin 1-mediated autophagy initiation [44]. Our study is consistent with this latter report and supports a positive role for ROCK1 in participating in doxorubicin-induced autophagy initiation through phosphorylating Beclin 1. These context dependent roles of ROCK in modulating autophagy are likely due to the complexity of autophagy regulation and also due to the variety of cellular functions controlled by RhoA/ROCK signaling. The roles and precise mechanisms downstream of ROCK1 in regulating autophagy during pathological cardiac remodeling triggered by other stresses require further investigation.

In summary, the present study demonstrated an important role for systemic and cardiomyocyte ROCK1 in doxorubicin cardiotoxicity, particularly in mediating autophagy dysregulation and apoptosis, which contribute to cardiac remodeling and dysfunction. Altogether, ROCK1 represents a potential therapeutic target in preventing the anti-cancer drug doxorubicin-induced heart failure and possibly other drug-induced cardiotoxicity related heart failure.

## MATERIALS AND METHODS

### Generation of mouse models

All animal experiments were conducted in accordance with the National Institutes of Health “Guide for the Care and Use of Laboratory Animals” and were approved by the Institutional Animal Care and Use Committee at Indiana University School of Medicine. Generation of ROCK1^−/−^ mice was as previously described [32].

Mice bearing a loxP-flanked ROCK1 allele (ROCK1^fl/fl^) were generated using the same genomic ROCK1 DNA clone containing exons 3-6 isolated from a 129 mouse genomic library as previously used to generate ROCK1^−/−^ mice [32]. In the targeting vector (Figure 4A), a PGK-Neo cassette is flanked by Frt sites and the gene segment containing the exon 5 is flanked by loxP sites. Deletion of exon 5 by Cre recombinase results in a frame-shift mutation in ROCK1, thus removing all residues from residue 137 to the end of the protein. Three independent ROCK1^fl-neo/+^ embryonic stem cell clones were identified and injected into C57BL/6 blastocysts to generate chimeric mice. The chimeric mice were bred with wild-type C57BL/6 mice for germline transmission of ROCK1^fl-neo^ allele. The genotypes of the offspring were identified by Southern blot analysis (Figure 4B) and PCR on DNA obtained from mouse tails. Mice used in the present study had been backcrossed to C57BL/6 for at least 8 generations. ROCK1^fl-neo/+^ heterozygous mice were then intercrossed to produce homozygous ROCK1^fl-neo/fl-neo^ mice. The presence of the Neo cassette has no detectable effect on the endogenous ROCK1 expression. ROCK1^fl-neo/fl-neo^ mice were then crossed with Gt (ROSA) 26Sor-Flp mice (Jackson Laboratory) to delete the Frt-flanked Neo cassette from the germ line. Offspring of these mice were heterozygous for the desired ROCK1^fl/+^ allele. To generate cardiomyocyte-specific ROCK1 knockout mice (MHC-Cre/ROCK1^fl/fl^), ROCK1^fl/fl^ mice were crossed to the MHC-Cre mice [41] and bred back to ROCK1^fl/fl^ mice (Figure 4C).

### *In vivo* mouse models of doxorubicin cardiomyopathy

Mice 8 to 9 weeks old were injected intraperitoneally with doxorubicin (Sigma) at 8 mg/kg or NS once weekly for 3 consecutive weeks, the cumulative dose being 24 mg/kg. Cardiac dimension and contractile performance were evaluated by noninvasive transthoracic echocardiography using a VisualSonics^®^ 2100 ultrasound machine for small animal imaging and MS400 transducer (Fujifilm Visual Sonics, Inc., Toronto, Canada) before each injection and 1 week after the last injection. Functional parameters of the left ventricle were measured using standard assessment techniques as previously described [33]. All mice were sacrificed after functional measurements; hearts were removed immediately, rinsed in pre-cooled PBS, and flash frozen in liquid nitrogen for later protein analysis, or cryopreserved in OCT medium for cryosections, or fixed in 4% paraformaldehyde buffer, followed by paraffin embedding.

To determine the early cytotoxic effects of doxorubicin *in vivo*, we have used a one-dose injury model. A single dose of doxorubicin at 10 mg/kg was administered intraperitoneally to mice 8 to 9 weeks old. Mice were sacrificed on day 2, 4, or 7 after treatment; the hearts were collected immediately as described above. To assess autophagic flux, mice received bafilomycin A1 at dose 1.5 mg/kg (Santa Cruz Biotechnology) intraperitoneally 2 hours before sample collecting. Heart homogenates were used for protein analysis by Western blot.

### Histology and quantitative analysis

Total heart weight was indexed to tibial length. Cryosections or paraffin sections were stained with hematoxylin/eosin for initial evaluation, picrosirius red/Fast green to identify collagen fibers, immunostaining for laminin to measure myocyte size, and TUNEL staining to monitor cardiomyocyte apoptosis as previously described [14, 33, 34, 36]. Myocyte diameter was measured using transnuclear width at the mid-ventricular level. The quantification of collagen-stained area was performed with Image-Pro software (Media Cybernetics). Apoptosis in mouse heart sections was assayed using the fluorescent ApopTag kit according to the manufacturer’s instructions (Chemicon). These sections were also counterstained with DAPI (Molecular Probes) and mounted with Vectashield. For all quantifications, a minimum of six sections from distinct regions of each heart sample and at least four hearts per group were analyzed.

### Electron microscopy analysis

Ventricular specimens (cubes less than 3 mm square) were fixed in 2.5% glutaraldehyde, underwent sectioning and heavy metal uranyl acetate staining for contrast by the Electron Microscopy Center of Indiana University School of Medicine. At least 6 separate sections from each animal strain were analyzed. Electron micrographs were acquired on a transmission electron microscope, Tecnai BioTwin (FEI) equipped with AMT CCD Camera (Advanced Microscopy Techniques).

### Protein analysis

Protein samples were prepared as previously described [14, 33, 36]. Ventricular tissue fragments were disrupted with a PYREX® Potter-Elvehjem tissue grinder on ice in lysis buffer containing proteinase and phosphatase inhibitors (Roche). The homogenate was centrifuged at 15,000 x *g* at 4°C for 15 minutes, and the supernatant was saved for immunoblotting. The blots were probed with primary antibodies to ROCK2 (sc-5561), FAK (sc-558) from Santa Cruz Biotechnology, ROCK1 (#4035), p-FAK-Tyr397 (#3283), Bax (#2772), LC3 (#2775), Beclin 1 (#3738), p62 (#5114) and p-AMPK-Thr172 (#2535) from Cell Signaling Technology, and p-Beclin1-Thr119 (ABC118) from MilliporeSigma. All blots were normalized to GAPDH (ABS16; MilliporeSigma) or to actin (MABT523; MilliporeSigma).

### Subcellular fractionation

Ventricular mitochondrial fractions were prepared as previously described [14, 36]. Samples with equal amounts of protein were analyzed by Western blot with specific antibodies. The purity of the mitochondrial fraction was assessed by Western blot analysis with GAPDH (a cytosolic protein) and cytochrome *C* oxidase subunit IV (COX IV) (a mitochondrial protein) (#4844; Cell Signaling Technology) as the markers, respectively.

### Statistical analysis

Data are reported as mean ± SE. Comparisons between groups were analyzed by Student’s *t*-test or ANOVA as appropriate, with *P* < 0.05 considered as significant.

## Abbreviations

MHC: myosin heavy chain
NS: normal saline
LVESD: left ventricular end systolic dimension
LVEDD: left ventricular end diastolic dimension
FS: fractional shortening
TUNEL: terminal deoxynucleotidyl transferase-mediated dUTP nick-end labeling
FAK: focal adhesion kinase
LC3: microtubule-associated protein 1 light chain 3
AMPK: AMP-activated protein kinase
SQSTM1: sequestosome 1
COX IV: cytochrome C oxidase subunit IV
